# Supplementary material for: Cloud BioLinux: pre-configured and on-demand bioinformatics computing for the genomics community
Source: BMC Bioinformatics. 2012 Mar 19;13:42. doi: 10.1186/1471-2105-13-42 (PMC3372431; doi:10.1186/1471-2105-13-42)
Supplement: Additional file 1 — Supplementary 1 Cloud BioLinux software documentation in the form of a mini, self-contained website. Users need to download and uncompress the .zip file, and open through a web browser the "index.html" file available on the main directory. (ZIP 1823 kb). [file 1471-2105-13-42-S1.ZIP › Cloud-BioLinux-Package-Documentation/docs/roche2gap.html]

Bio-Linux Software Documentation Pages

Back to search form

## roche2gap

|  |  |
| --- | --- |
| Name | roche2gap |
| Description | **roche2gap** is a script that converts 454 ace format assemblies to gap4 format, allowing viewing and editing using Staden's gap4 assembly editor.  roche2gap comes in the bio-linux-assembly-conversion-tools package.  Further details on how to use this software can be found by referring to the remote documentation.  Other tools available on Bio-Linux for handling data from new sequencing technologies are described in the bioinformatics documentation on the NEBC website. |
| Homepage | http://genome.imb-jena.de/software/roche454ace2caf/ |
| Remote Documentation | http://nebc.nox.ac.uk/tools/bioinformatics-docs/high-throughput-seq/assembly-viewing#staden |
